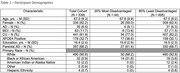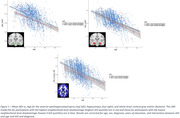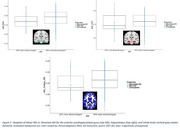# Associations between Neighborhood‐Level Disadvantage and Neuroimaging Markers of Microstructural Neurodegeneration Across the Alzheimer’s Disease Continuum: A Mixed‐Longitudinal Study

**DOI:** 10.1002/alz.095517

**Published:** 2025-01-09

**Authors:** Jason F Moody, William R. Buckingham, W. Ryan Powell, Douglas C Dean, Andrew L Alexander, Amy J.H. Kind, Barbara B. Bendlin

**Affiliations:** ^1^ Wisconsin Alzheimer’s Disease Research Center, University of Wisconsin‐Madison, School of Medicine and Public Health, Madison, WI USA; ^2^ Health Services and Care Research Program, University of Wisconsin‐Madison, School of Medicine and Public Health, Madison, WI USA; ^3^ Department of Pediatrics, University of Wisconsin‐Madison, Madison, WI USA; ^4^ Department of Medical Physics, University of Wisconsin‐Madison, Madison, WI USA; ^5^ Waisman Center, University of Wisconsin‐Madison, Madison, WI USA; ^6^ Department of Psychiatry, University of Wisconsin‐Madison, Madison, WI USA; ^7^ Geriatric Research Education and Clinical Center, Middleton Memorial VA Hospital, Madison, WI USA; ^8^ Wisconsin Alzheimer's Disease Research Center, University of Wisconsin‐Madison, School of Medicine and Public Health, Madison, WI USA

## Abstract

**Background:**

Residence in highly socioeconomically disadvantaged neighborhoods has recently been associated with Alzheimer’s disease (AD) neuropathology at autopsy, cognitive decline, and magnetic resonance imaging (MRI) markers of volumetric brain atrophy in cognitively unimpaired adults. Furthermore, there is mounting evidence that markers of brain microstructure derived from diffusion‐weighted MRI (DWI), including neurite density index (NDI), orientation dispersion index (ODI), and isotropic volume fraction (ISO), are sensitive to AD‐related neurodegeneration.

In this study, we used linear mixed‐effects (LME) modeling to investigate the hypothesis that neighborhood‐level disadvantage is associated with mixed‐longitudinal trajectories of microstructural neurodegeneration in 539 late‐middle‐aged participants across the AD continuum.

**Methods:**

539 participants (Table 1) from the Wisconsin Registry for Alzheimer’s Prevention and the Wisconsin Alzheimer’s Disease Research Center were imaged between 1 and 5 times with multi‐shell DWI (constituting 865 total scans). For each scan, average NDI, ODI, and ISO values were extracted from the hippocampus, anterior parahippocampal gyrus, and whole brain cortical gray matter. Geocoded participant addresses were linked to neighborhood disadvantage as measured by the Area Deprivation Index, a marker derived from 17 census indicators of education, employment, poverty, and housing quality. Statewide ADI was binarized for each participant (the highest quintile/most disadvantaged or lowest 4 quintiles/least disadvantaged) and used as a predictor of DWI metrics in LME models with age, sex, diagnosis, education level, and interactions between ADI and age and ADI and diagnosis as covariates.

**Results:**

NDI—a measure sensitive to axonal and dendritic loss—was significantly lower (denoting more neurodegeneration) in the most disadvantaged group in all three assessed brain regions (P_Corrected_ < 0.05), and there was a significant interaction between ADI and age (P_Corrected_ < 0.05) for hippocampal NDI (Figures 1‐2). No other ADI‐DWI associations or ADI interactions survived Benjamini‐Hochberg correction for multiple comparisons.

**Conclusion:**

Our findings suggest that living in a disadvantaged neighborhood is associated with neuronal degeneration, evidenced by the apparent loss of neurites, and that this process accelerates with age across the AD continuum. As a result, addressing disparities in the social determinants of health may have the potential to reduce the likelihood of neuronal injury in aging adults.